# Effect of pH and Pea Protein: Xanthan Gum Ratio on Emulsions with High Oil Content and High Internal Phase Emulsion Formation

**DOI:** 10.3390/molecules26185646

**Published:** 2021-09-17

**Authors:** Eliana Marcela Vélez-Erazo, Karina Bosqui, Renata S. Rabelo, Miriam Dupas Hubinger

**Affiliations:** 1 Department of Food Engineering and Technology, School of Food Engineering, University of Campinas, Monteiro Lobato Street, 80, Campinas 13083-862, Brazil; karinaboqui@gmail.com (K.B.); mhub@unicamp.br (M.D.H.); 2 Brazilian Synchrotron Light Laboratory (LNLS), Brazilian Center for Research in Energy and Materials (CNPEM), Campinas 13083-970, Brazil; rerabelo.eng@gmail.com

**Keywords:** stability, oil loss, oil structuring, HIPE, electrostatic interaction, layer-by-layer

## Abstract

Electrostatic interaction between protein and polysaccharides could influence structured liquid oil stability when emulsification is used for this purpose. The objective of this work was to structure sunflower oil forming emulsions and High Internal Phase Emulsions (HIPEs) using pea protein (PP) and xanthan gum (XG) as a stabilizer, promoting or not their electrostatic attraction. The 60/40 oil-in-water emulsions were made varying the pH (3, 5, and 7) and PP:XG ratio (4:1, 8:1, and 12:1). To form HIPEs, samples were oven-dried and homogenized. The higher the pH, the smaller the droplet size (Emulsions: 15.60–43.96 µm and HIPEs: 8.74–20.38 µm) and the oil release after 9 weeks of storage at 5 °C and 25 °C (oil loss < 8%). All systems had weak gel-like behavior, however, the values of viscoelastic properties (G′ and G″) increased with the increment of PP:XG ratio. Stable emulsions were obtained at pHs 5 and 7 in all PP:XG ratios, and at pH 3 in the ratio 4:1. Stable HIPEs were obtained at pH 7 in the ratios PP:XG 4:1, 8:1, and 12:1, and at pH 5 at PP:XG ratio 4:1. All these systems presented different characteristics that could be exploited for their application as fat substitutes.

## 1. Introduction

Edible oil structuring has received considerable attention from the food industry and scientific community as a potential alternative of replacing saturated fats with healthier alternatives such as liquid polyunsaturated oil. Oil structuring aims at incorporating as much oil as possible in a stable matrix, using a reduced number of structuring agents. Traditionally, oil structuring has been made by direct gelation using lipid additives such as monoacylglycerides, triacylglycerides, different types of waxes, etc., to promote the gelling of the oil, obtaining an oleogel [1,2,3,4]. Food-grade biopolymers such as proteins and polysaccharides have been used as structuring agents in an indirect approach [5,6,7].

The use of hydrocolloids for this purpose has some advantages compared with the direct structurants because hydrocolloids are accepted and known by the food industry, being considered Generally Recognized as Safe (GRAS) by the Food and Drugs Administration (FDA), and avoiding regulatory restrictions and being these of easy access. Additionally, they have a lower cost than direct structurants, and, when used in the indirect approach, moderate temperatures are applied during the oil structuring process. For these reasons, hydrocolloids have been used to structure systems that will replace saturated fats, obtaining final products with similar characteristics to the original products [8].

Emulsification is an indirect methodology applied to liquid oil structuring. In this process, the interfacial functional property of proteins is exploited to form oil in water emulsions, while the gelling or thickening property of polysaccharides is exploited to stabilize the formed emulsion sterically. The objective of emulsification in oil structuring is to reach a semi-solid system with the formation of emulsions (oil ~60%), emulgels (by mixing hydrogels with emulsions), or High Internal Phase Emulsion (HIPE) (oil > 74%). These systems have been widely used in pharmaceutical industries as novel drug delivery of hydrophilic and lipophilic bioactive compounds [3,9,10], or as substitutes of saturated fats in food formulations, due to their rheological and mechanical characteristics [11,12].

The interactions between protein and polysaccharide are involved in many processes affecting food products’ mechanical, sensorial and functional properties [13]. Electrostatic interaction between these polymers occurs at pHs below protein’s isoelectric point. At this pH, protein has a positive charge, and the majority of polysaccharides have a negative charge [14], and electrostatic interaction is possible between them. Pea protein (PP) is a vegetable protein with an isoelectric point between pH 3.84–4.23 [15], and has been of great interest for alternative applications to animal protein. On the other hand, the food industry uses xanthan gum (XG) mainly as a thickening agent. This polysaccharide is known for its negative charge due to the presence of pyruvic acid residue and an acetyl group attached to the main chain [16].

The electrostatic interaction between PP and XG could be used in two ways as an emulsion stabilizer. The first way is the layer-by-layer technique, which has been used to improve emulsion stability, especially in emulsions with low oil content [17,18]. In this process, at first, an emulsion is formed by the protein at a pH below the isoelectric point, and then, the polysaccharide is deposited electrostatically in the interfacial protein layer [19]. The second way is the formation of polyelectrolytic complexes between the protein and polysaccharide before the emulsification process. 

In oil structuring application, some works have used the complexes formation to form and stabilize the emulsion, i.e., electrostatic complexes of soy protein/κ-carrageenan [20], whey protein isolate/pectin [21,22], PP/pectin [23], modified starch/chitosan [24] or egg yolk-modified starch [25]. Some of these studies called their systems high internal phase Pickering emulsions (HIPPEs) because colloidal particles stabilize the emulsions at low pH. Nevertheless, it is necessary to study other ways to use the electrostatic interaction (layer by layer technique) where the stabilization mechanism is not using colloidal particles, and also, compare the HIPEs’ stability when the electrostatic interaction is not promoted.

A previous work of our research group [26] aimed to select the best polysaccharide to stabilize emulsions and HIPEs without the pH modification at a fixed PP:XG ratio. In that work, it was found that XG can stabilize HIPEs after the emulsion drying. Considering that some studies used electrostatic complexes as HIPE stabilizers with promising results, the objective of this study was to promote the electrostatic interaction between PP and XG by the layer-by-layer technique verifying if more stable systems than those produced at neutral pH were generated, and what was the role of the PP:XG ratio in the physical characteristics of the final system.

In this sense, sunflower oil was structured forming emulsions with 60% of oil and HIPEs, using PP and XG as structurants at different PP:XG ratios and pHs to promote, or not, the electrostatic deposition of XG in the emulsion stabilized by PP. Emulsions and HIPEs were characterized by their micro and macrostructure; droplets mean diameter and size distribution, rheological behavior, and stability against oil loss were studied for 9 weeks at 5 and 25 °C.

## 2. Results and Discussion

### 2.1. Hydrocolloids and Pea Protein: Xanthan Gum Electrostatic Complexes’ Characterization

**Figure 1:**
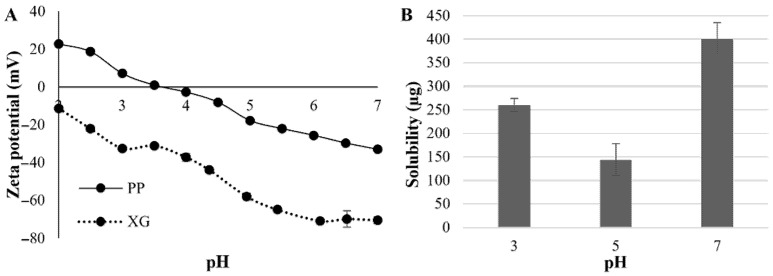
Zeta potential of pea protein (PP) and xanthan gum (XG) (**A**) and Protein solubility (**B**).

The results for zeta potential of the pea protein and xanthan gum are shown in Figure 1A. The zeta potential of the XG decreased with increasing pH until they reached values close to −70 mV, which is in accordance with observed by Wang et al. [27]. On the other hand, the PP has a positive zeta-potential below pH 3.5–4.0 and negative above this pH value, which is in accordance with the literature, that reported the isoelectric point of PP between pH 3.84 and 4.23 [15].

The solubility of pea protein at pH 3, 5 and 7 are shown in Figure 1B. The highest protein solubility is observed at the highest pH (7) with 400 µg of protein/mL of water. Regarding pHs 3 and 5, lower solubility values were observed, leading to the belief that the isoelectric point of the protein could be between these pHs. Johansson et al. [28] reported similar values of pea protein solubility (pH 3: 395 μg/mL, pH 4.2: 194 μg/mL and pH 7.4: 595 μg/mL).

Since the isoelectric point of PP was under pH 4, and XG was totally soluble at all evaluated pHs (data not shown), PP/XG electrostatic interaction was promoted at pH 3 by the electrostatic deposition of XG in a PP-stabilized pre-emulsion. In general, the role of PP in the system is the emulsion formation due to its amphiphilic structure that permits its location in the oil/water interphase. Regarding the XG role, it promotes steric stabilization modifying the viscosity of the continuous phase. Due to the possible electrostatic interaction (at pH 3) or repulsion (pH 5 and 7) between the biopolymers, three biopolymer ratios were also studied in the formation of 60% sunflower oil emulsions and HIPEs.

### 2.2. Macrostructure and Water Loss 

**Figure 2:**
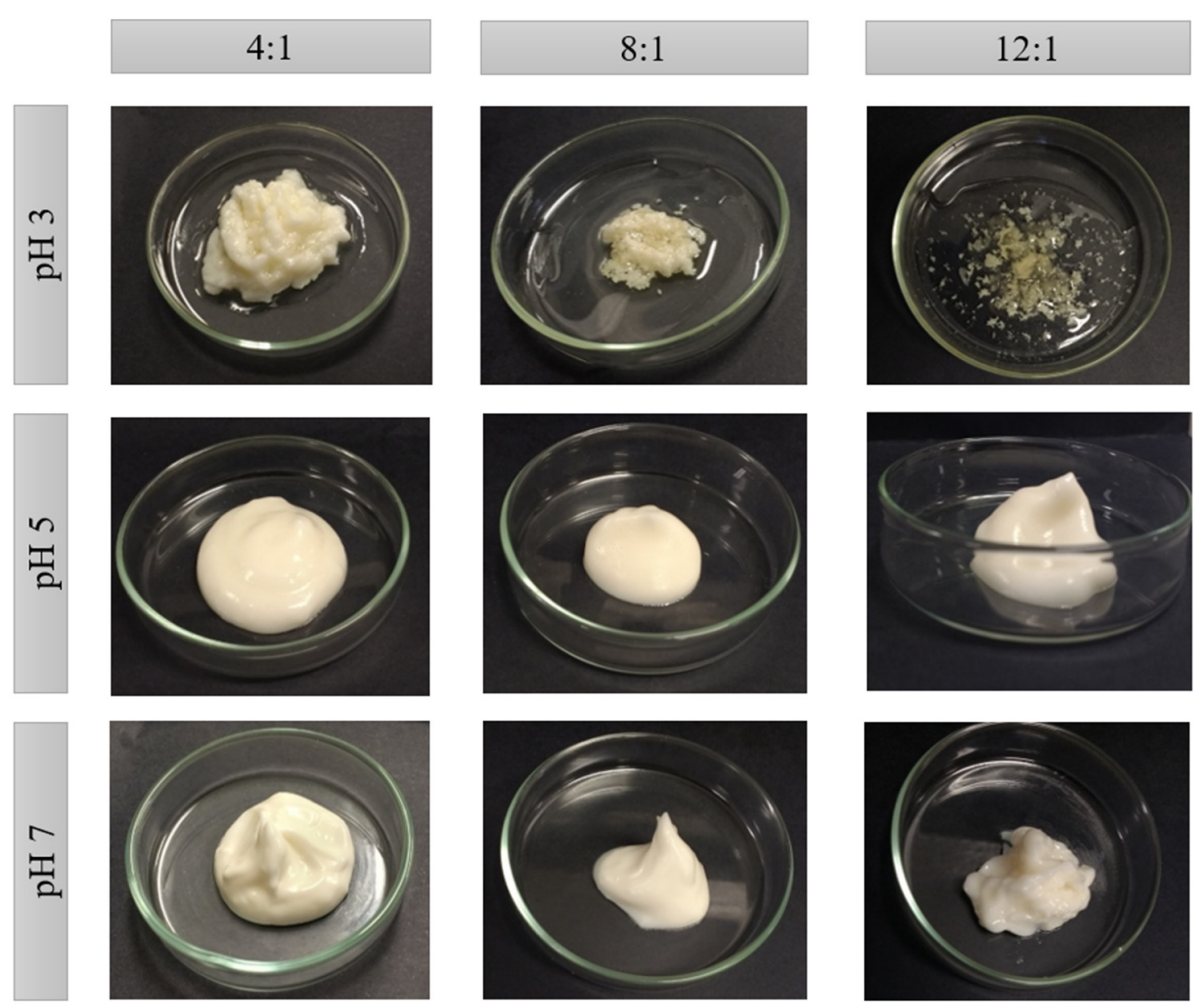
HIPEs macroscopic appearance obtained with pea protein (PP) and xanthan gum (XG) at different PP:XG ratios (4:1, 8:1 and 12:1) and pHs (3, 5 and 7).

**Figure A1:**
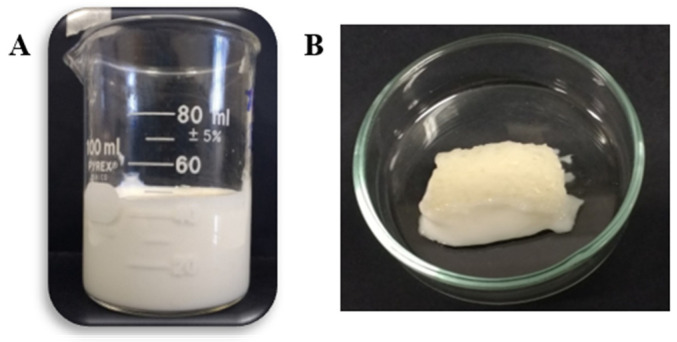
(**A**): Emulsion, (**B**): dried product before homogenization.

The emulsions were visually uniform in all conditions after preparation (zero time) (Appendix A Figure A1A). Subsequently, the emulsions were dehydrated (Appendix A Figure A1B) and gently homogenized, resulting in the systems in Figure 2. These dehydrated systems are called High Internal Phase Emulsions (HIPEs) due to the high oil content trapped into them (>73%).

The instability of the HIPEs formulated at pH 3 in Figure 1 is easily observed. At this pH, the bigger the PP:XG ratio, the less the amount of water retained (1.24–5.87 g), and the greater the oil released (Table 1), which is directly associated with the higher water holding capacity of XG when compared to PP. This instability is attributed to the strong electrostatic interaction between PP and XG. It is possible that during the drying process, the strength of the electrostatic interaction between the biopolymers would be too much, promoting the formation of PP:XG electrostatic complexes and the subsequent expulsion of water and oil from the polymeric structure. 

On the other hand, the formulations obtained at pH 5 and 7 showed a cream arrangement and no visible oil release was observed in these systems. The visual structure seems smoother and creamier at pH 5 than pH 7, mainly when the PP:XG ratio decrease (Figure 1). This behavior is related to the lower mass loss (water) during the drying process. In this case, the electrostatic repulsion between biopolymers results in stable systems because XG keeps dispersed in the continuous phase. Additionally, HIPEs at pH 5 presented more water holding than HIPEs at pH 7 (between 17.41–26.41% and 11.55–21.67% for pH 5 and 7, respectively), decreasing the water holding as the PP:XG ratio increased. A detailed assessment of the water loss for all systems is presented together with the mass balance of the dehydration process in Table 1.

When comparing the emulsion stability with protein solubility, it was observed that a lower solubility of the protein is not necessarily related to the lower stability of the emulsion. In this case, the electrostatic interaction between biopolymers at pH 3 had more influence on the formation of unstable systems than the low solubility of the protein at pH 5. On the other hand, it is possible that the low solubility of the protein at pH 5 may have affected the type of structure formed, compared to systems at pH 7. 

A similar macrostructure was observed by other authors in emulsions with 80% oil stabilized by whey protein isolate and pectin electrostatic complexes [21]. The authors also reported an agglomerated structure at low pH (3.5) and a creamy structure at pH above the isoelectric point. Jiao et al. [29] and Hao et al. [30] presented the same creamy structure in HIPEs stabilized by microgels (Pickering) of peanut protein isolate and soy glycinin, respectively.

### 2.3. Microstructure and Droplet Size

**Figure 3:**
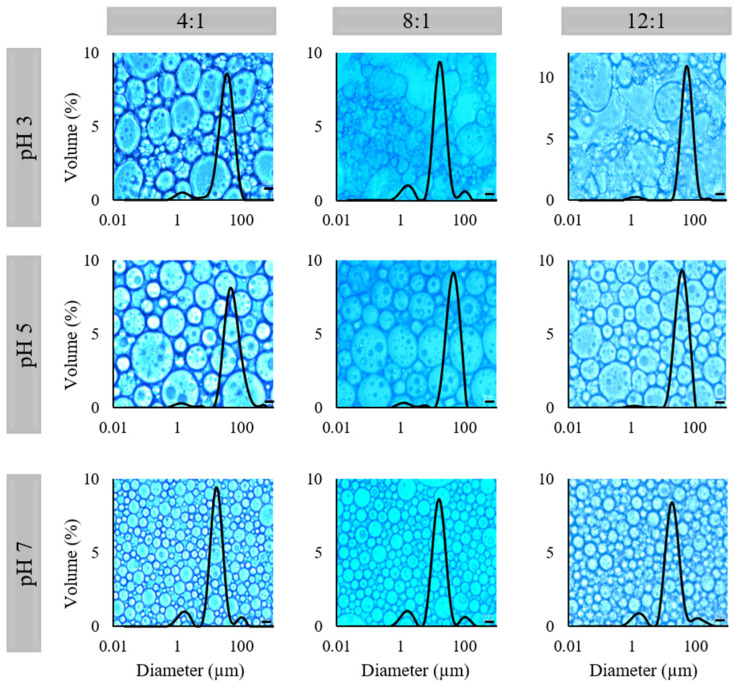
Micrographs and droplet size distribution of emulsions produced at pea protein: xanthan gum (PP:XG) ratio of 4:1, 8:1, and 12:1; and pH 3, 5, and 7. Bar scale: 10 µm.

In Figure 3 and Table 2 the micrographs with the droplet size distributions and the values of the mean diameters of the emulsions can be observed. All systems presented a polymodal droplet size distribution with a very prominent peak and low Span value, explaining the low polydispersity in the emulsions droplet size.

At pH 3, only the PP:XG ratio 4:1 presented the characteristic microstructure of an emulsion (Figure 3). The electrostatic deposition of XG on the PP-stabilized emulsions promoted emulsion stability, avoiding the droplets’ coalescence. At PP:XG ratios 8:1 and 12:1 exists a protein excess, occurring bridging flocculation because there is insufficient polysaccharide to cover the protein surface [31]. Even without a defined emulsion structure and free oil presence in the micrograph, no phase separation was observed in PP:XG ratio 8:1 and 12:1 emulsion. It is possible that the xanthan gum layer may help the emulsion stability by steric forces, and for this reason, no oil release was observed.

**Figure 4:**
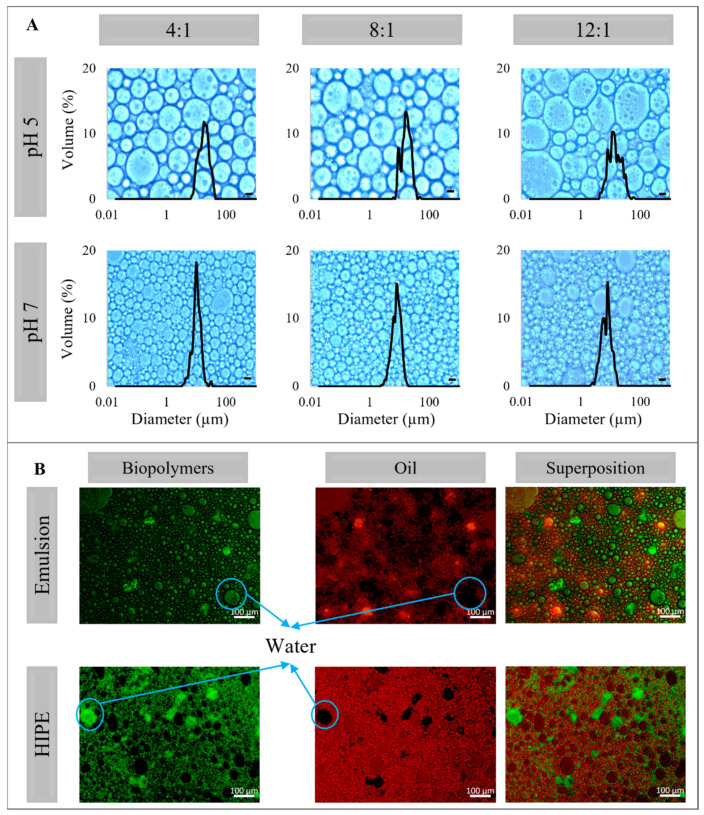
(**A**) Micrographs and droplet size distribution of HIPEs produced at pea protein: xanthan gum ratio of 4:1, 8:1, and 12:1; and pH 5 and 7. Bar scale: 10 µm. (**B**) Fluorescence microscopy of emulsion and HIPE at 4:1 and pH 7.

At pH 5 and 7, all the emulsions presented emulsion structure with droplets significantly bigger at pH 5 than at pH 7 (Figure 3). The droplet size of emulsions at pH 5 varied from 40.60 to 51.79 µm, and at pH 7 varied from 19.17 to 23.26 µm (Table 2). The D_[4,3]_ was chosen to characterize the emulsions due to their similarity with the mode of the size distribution. At pH 7 PP is more soluble than at the other pHs studied (Figure 1B). The more soluble the protein, the less the protein–protein interaction and the greater was the availability of hydrophobic groups to come into contact with sunflower oil, and therefore, the smaller the droplet size observed. Additionally, at pH 5 and 7 both biopolymers, PP and XG, present negative charges (−18 and −60 mV at pH 5, and −33 and −70 mV at pH 7, respectively, Figure 1A). This behavior promotes the electrostatic stabilization of the emulsion by the electrostatic repulsion, avoiding the droplets interaction and future destabilization. Finally, the emulsion at pH 3 and PP:XG ratio 4:1 presented an intermediate mean diameter (34.28 µm). No clear effect of PP:XG ratio on the mean diameter was observed. On the other hand, the microstructure of the HIPEs observed in Figure 4A is similar to their correspondent emulsion.

The diameters calculated from the micrographs (D_[4,3]_, D_[Th]_, and M_o_) are lower than the diameters observed in the emulsions (Table 2). This difference is attributed to the method used to measure the HIPEs’ mean diameter (micrograph analyses). This manual method was used because it was impossible to disperse the HIPEs in the Mastersizer equipment due to their creamy and compact structure. D_[Th]_ characterizes better than D_[4,3]_ the HIPEs since D_[Th]_ diameter is closer to M_o_. HIPEs at pH 5 presented a mean diameter between 16.60 and 20.38 µm, and those at pH 7 were between 8.00 and 11.46 µm. No PP:XG ratio effect was observed on the D_[Th]_. Yi et al. [23] and Feng et al. [32] reported similar mean droplet diameter (38.3–55.0 µm and 11.2–25.45 µm, respectively) and similar micrographs in HIPPEs stabilized with PP:pectin colloidal particles.

Figure 4B shows the fluorescence microscopy for the emulsion and HIPE at 4:1 PP:XG ratio and pH 7. Green and red microscopies indicate polymers and oil presence, respectively, and in the last one, the micrographs superposition can be observed. All systems presented high dispersed phase density with the structure of oil-in-water emulsions. Black areas in the oil micrographs indicate water presence, some of which have polymer presence, especially in the HIPE microscopy, indicating that the drying process did not affect the emulsion structure.

### 2.4. Rheological Analysis

**Figure 5:**
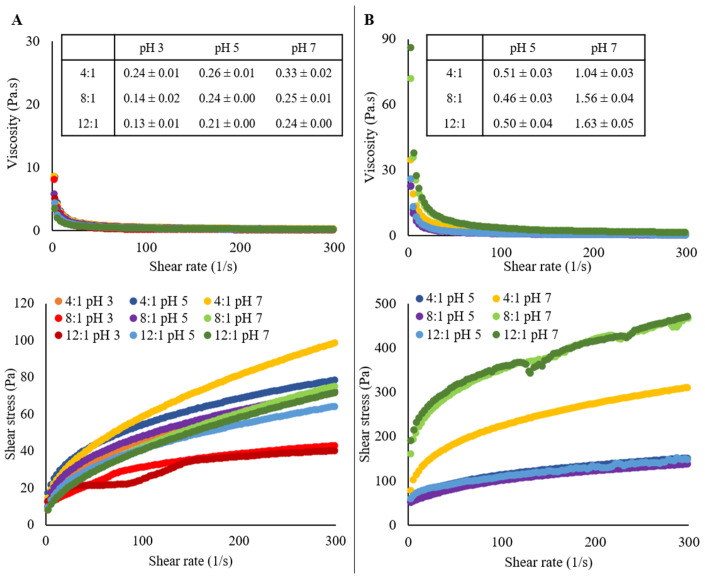
Steady-state rheology (Shear stress and viscosity) of the emulsions (**A**) and HIPEs (**B**). Apparent viscosity (Pa·s) at a shear rate of 300 1/s is presented in the tables.

Flow curves (shear stress and viscosity) of the emulsions and HIPEs are presented in Figure 5.

Herschel–Bulkley model was used to fit the rheological parameters of the flow curves, presenting a high correlation (r^2^ > 0.98) to shear-thinning behavior (n between 0.34 and 0.63 for emulsions and 0.32 and 0.58 for HIPEs). This behavior was also reported by other authors, in which HIPEs were stabilized by PP:pectin electrostatic complexes [32] and PP with different polysaccharides without pH modification [26]. Figure 5A shows the shear stress and apparent viscosity curves for emulsions. Systems at pH 3 with ratios 8:1 and 12:1 presented the lowest shear stress values and apparent viscosities at 300 1/s of shear rate (0.14 and 0.13 Pa·s, respectively). This could be related to the free oil observed in their micrographs when XG’s thickening properties avoided the oil expulsion and subsequent phase separation.

On the other hand, emulsion at pH 7 with a ratio of 4:1 presented the highest apparent viscosity (0.33 Pa·s). A relationship between the droplet size and the viscosity can be found, since the higher the mean droplet size, the lower the surface area to droplets interaction, and finally, the smaller the shear-thinning behavior and viscosity [33], as can be seen for emulsions at pH 3 with ratios 8:1 and 12:1. In the same way, HIPEs viscosities at pH 5 were lower than at pH 7 (between 0.46–0.51 and 1.04–1.63 Pa·s, respectively) (Figure 5B).

Similar to viscosity, the initial shear stress (σ_o_) also presented a difference when emulsion and HIPEs are compared. Emulsions’ σ_o_ varied between 2.89 to 12.09 Pa, while HIPEs varied from 31.9 to 57.7 Pa for pH 5, and from 23.8 to 176.8 for pH 7. This difference is due to the drying process. The higher the water loss, the higher the σ_o_ and viscosity, being the HIPE at pH 7 with a ratio of 12:1 the stronger shear-thinning fluid with a more cohesive structure, as can be corroborated in Table 1 and Figure 2.

The decrease in viscosity and the shear-thinning behavior with pH decrease is related to the PP-XG electrostatic interaction at 4:1, and PP-PP electrostatic interaction at 8:1 and 12:1, the last two due to protein excess in the system. This viscosity decrease indicates polymer interfacial accumulation due to its electrostatic interaction [20]. At pH 7, no electrostatic interaction is promoted, so the XG and the PP in excess increase the continuous phase’s viscosity.

**Figure 6:**
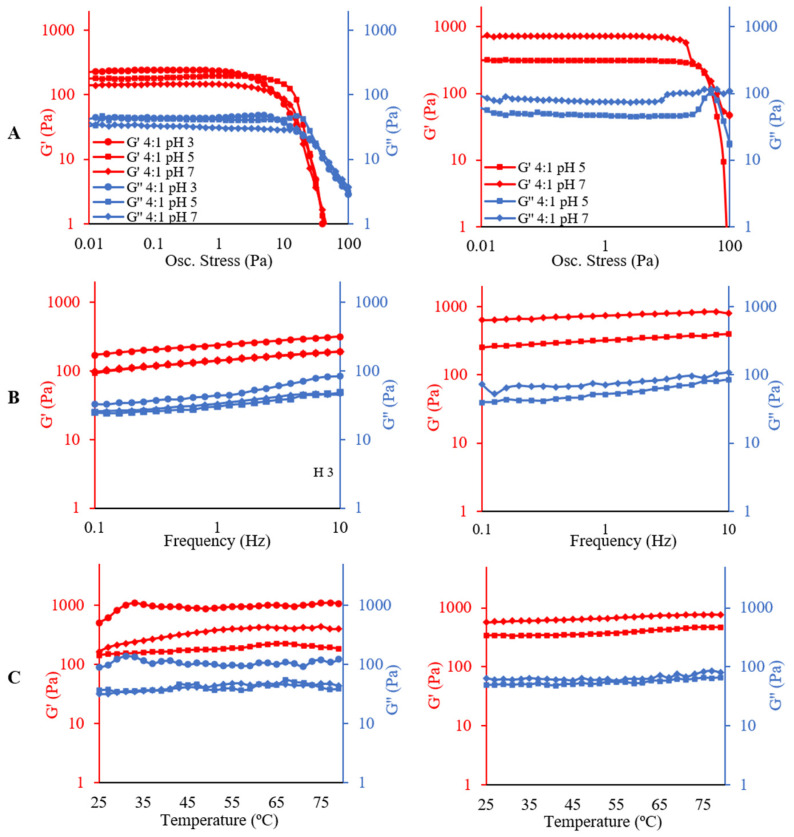
Amplitude (**A**), frequency (**B**) and temperature (**C**) curves of pea protein and xanthan gum-stabilized emulsions (**left**) and HIPEs (**right**).

Regarding oscillatory assays, Figure 6 shows the amplitude, frequency, and temperature curves for emulsions on the left side and HIPEs on the right side. The oscillatory rheology results present only the variation of pH at a ratio of PP:XG 4:1 to not crowd the emulsion figures.

In the stress sweeps analysis (Figure 6A), it is possible to determine two regions, the linear viscoelastic region (LVR) where the storage modulus (G′) and the loss modulus (G″) are constant, and the nonlinear region, where G′ and G″ decrease. Using this information, it is possible to analyze some rheological parameters in the LVR such as G′_LVR_, G″_LVR_, and loss-tangent (tan δ_LVR_), as well as the limiting value of oscillatory stress (OS_L_), the flow-point stress (FP_S_) and the flow-point G value (FP_G_) [34]. These parameters are shown in Table 3 for all experiments.

The G′_LVR_ and G″_LVR_ values for emulsions varied between 98 and 254 Pa, and between 22 and 43 Pa, respectively, increasing with both pH and PP:XG ratio decreasing. The ratio between G′_LVR_ and G″_LVR_ can be observed by Tan δ_LVR_. As shown in Table 3, this parameter is between 0.15 and 0.32, indicating the predominance of elastic behavior at the LVE region. At pH 3, the highest G′ value was observed, especially at 8:1 and 12:1 PP:XG ratios, which presented a high limiting value of oscillatory stress (OS_L_) (2.17 and 2.72 Pa) and the highest stress at flow point (FPs) (62.99 and 50.10 Pa). OS_L_ indicates the maximum stress applied before structural changes start, and the flow point indicates the stress at which the first non-linear changes in the structure occur (G′ = G″) [34]. This behavior identifies the 8:1 and 12:1 PP:XG emulsions as the most strong emulsions, since strong gels may remain in the linear viscoelastic region at greater strains than weak gels [35].

The highest OS_L_ was observed for emulsion at pH 5 and 8:1 PP:XG (3.57 Pa); however, with increasing stress, a sharper downturn in G′ value was observed than those for emulsion at pH 3 and 8:1 and 12:1 PP:XG. The G′ decrease is associated with the deformability [35]; a larger G′ drop when stress is increased means that the structure does not break homogeneously under shear, characteristic of non-creamy systems.

For HIPEs systems a considerable increase in G′_LVR_ and G″_LVR_ was observed, which varied between 311 and 1183 Pa for G′_LVR_ and between 43 and 105 Pa for G″_LVR_, increasing with pH increase. Tan δ_LVR_ for these systems are lower than for emulsion (values from 0.08 to 0.17), showing a stronger difference between G′_LVR_ and G″_LVR_ regarding elastic behavior. This behavior can be correlated with the macroscopic appearance and the water content (Figure 1 and Table 1), especially for PP:XG 1:8 and 1:12 at pH 7, which have a more visual solid-like structure and less water content.

HIPEs OS_L_, FPs and FP_G_ increased too when compared with emulsions. OS_L_ varied from 2.25 to 14.22 Pa, FPs from 50.11 to 86.25 Pa and FP_G_ from 76 and 108 Pa. These characteristics made HIPEs stronger gels than emulsions. On the other hand, HIPEs at PP:XG 1:8 and 1:12 at pH 7 remained stable during all oscillatory stress sweeps, being the most stable systems.

At high oscillatory stress, it can be observed that at pH 5 for both emulsions and HIPEs, a peak in G″ value is formed near to flow point (Figure 6A). The beginning of the peak formation is related to the occurrence of micro-cracks in the structure. When the maximum peak point is reached, the flow point is observed, and macro-cracks are formed throughout the sample. After that, G″ > G′ and the system shows a fluid behavior.

An oscillatory stress of 0.1 Pa was chosen to perform the frequency and temperature sweeps since this value was within the LVR for all samples (emulsions and HIPEs), and it is expected that the weak gel network will not be modified.

Frequency sweep analysis shows how the viscous and elastic behavior changes when the frequency is increased, being useful to distinguish between entanglement network, weak gel and strong gel [36]. Figure 6B shows that within the range of the studied frequency, both emulsions and HIPEs present the storage modulus (G′) larger than the loss modulus (G″), predominating a solid-like behavior for all samples, where the deformations are elastic and recoverable.

All systems (emulsions and HIPEs) have a typical week gel-like behavior. Both modulus G′ and G″ have little frequency dependence, without significant changes of G″ through frequency (strong gels), and no crossover point occurred (entanglement network) [36]. However, emulsions at pH 3 presented the highest gel behavior (long distance between G′ and G″) and did not achieve a uniform structure after drying and homogenization. At pH 5 and 7, no interaction occurs between PP and XG and a more flexible structure is achieved; this allows concluding the system would support the drying and homogenization processes.

On the other hand, as expected, an increase in G′ and G″ values were observed for HIPEs, being higher at pH 7 than at pH 5. This increase can be related to the formation of a more complex structure due to the drying process. At pH 7, a small increase in G′ and G″ was observed with PP:XG ratio increase; however, this change was not observed at pH 5. These results are in accordance with the structures observed in Figure 1, where the biggest change in the macrostructure is observed at pH 7 than at pH 5.

Finally, emulsions and HIPEs were subjected to temperature sweeps to study their thermal stability (Figure 6C). This was performed to determine the sensitivity of the structure of emulsions and HIPEs to thermal changes. All samples were thermostable between 25 and 80 °C, since the structure had not changed when subjected to temperature changes.

Other authors found similar results for oscillating stress, frequency, and temperature sweep for emulsions with 60–75% oil and oleogels, also obtained by emulsion dehydration [37,38,39,40,41].

### 2.5. Oil Release

**Figure 7:**
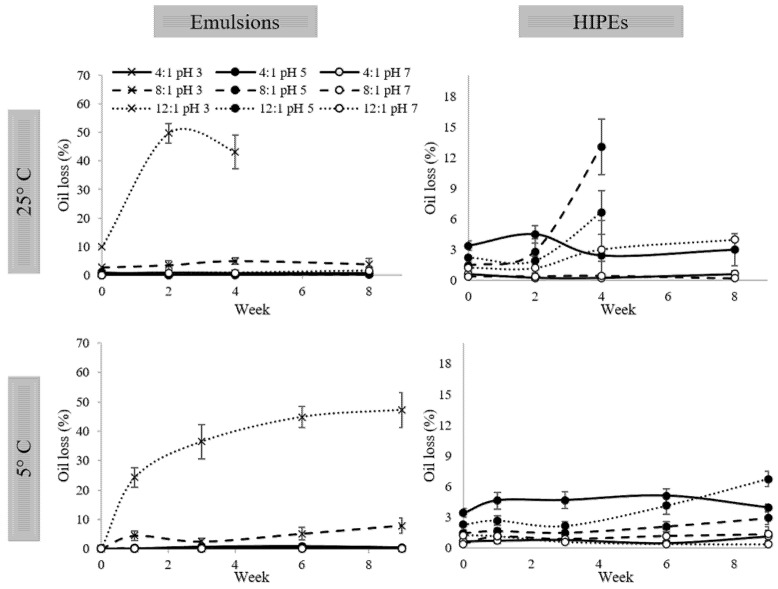
Oil loss of emulsions and HIPEs stored at 5 and 25 °C.

Emulsions and HIPEs stability were studied through oil release, during 8 weeks of storage at 25 °C and 9 weeks at 5 °C (Figure 7).

In general, it can be observed that all emulsions are stable at both temperatures showing oil release below 2%, except for the emulsion at PP:XG ratio of 8:1 at pH 3, which shows oil release below 8% during the storage; nevertheless, it was stable too. Only emulsion at PP:XG ratio of 12:1 at pH 3 was not stable, since a notable increase in the oil release was observed along the storage time at both temperatures, 5 and 25 °C. In this case, at 25 °C of storage, when ~50% of oil release was achieved, the structure was lost, and it was not possible to continue with the measurements. The biggest oil release of emulsions at PP:XG ratios of 8:1 and 12:1 at pH 3 is related to the non-defined emulsion microstructure observed in Figure 2, leaving the oil more available to be expelled during structural changes that occur during storage.

Regarding the HIPEs storage at 25 °C, samples at PP:XG ratios of 8:1 and 12:1 at pH 5 presented the worst stability since, at week 4, a maximum amount of oil (13% and 7%, respectively) was expulsed, and after this time, the structure was lost, and it was not possible to continue with the analysis. All the other HIPEs were stable during storage at 25 °C. HIPEs at PP:XG 12:1 pH 7 and 4:1 pH 5 presented oil release of ~4%, and the more stable HIPEs were those at PP:XG 8:1 and 4:1 at pH 7, which presented oil release below 1% during 9 weeks.

At 5 °C of storage, a similar behavior to 25 °C was observed. HIPE at PP:XG 12:1 pH 5 presented an increasing tendency in oil release after week 3, passing from 2% to 7% and showing a tendency to destabilize. All the other HIPEs were stable with oil release below 5%. Finally, the most stable HIPEs were those at pH 7 at any ratio, showing oil release below 1% during the 9 weeks of storage.

The stability of emulsions and HIPEs against phase separation at different pHs is related to the microstructure observed in each system. The higher the pH, the smaller the droplet size (Figure 3 and Figure 4) and, in consequence, the better the stability during the storage time.

The use of proteins with polysaccharides in the oil structuring had demonstrated that this approach shows promising results in the long-time stability of structured systems. Other authors also have reported stability in their systems. Hao et al. [30] presented HIPEs stabilized by soy glycinin and soy polysaccharides. The systems were stable for 30 days without changes in the visual appearance and droplet size, even with heat treatment before the characterization. Vélez-Erazo et al. [26] obtained stable emulsions (60% of oil) stabilized by pea protein and carrageenan and HIPEs (>73% of oil) stabilized by PP and tara gum or xanthan gum stored at 25 °C for 4 weeks (oil release less than 2%). Feng et al. [32] also worked with PP in electrostatic complexes with pectin obtaining stable systems for 30 days with a slight droplet size increase. Huang et al. [42] produced HIPEs stabilized by nanocomplexes at different chitosan–casein phosphopeptides ratios. All systems were stable to phase separation for 6 months at room temperature with small changes in the droplet size. The authors concluded that structured oils made with an emulsion approach could be used as oral delivery systems of nutraceuticals.

## 3. Materials and Methods

### 3.1. Material

As structurants, pea protein (PP) (86% protein, CAS 90082-41-0, R&S Blumos Industrial e Comercial Ltd.a (Cotia, Brazil)) and xanthan gum (XG, CAS 11138-66-2, Danisco São Paulo, Brazil) were used. Nile red and fluorescein 5(6)-isothiocyanate (FITC) (Sigma Aldrich, Wicklow, Ireland) were used as dyes. Sunflower oil (SO) was purchased at a local market. To adjusting pH phosphoric acid (Dinâmica Química Contemporânea Ltd.a, Indaiatuba, Brazil) and sodium hydroxide (J.T. Baker, Xalostoc, Mexico) were used. All assays were made with Milli-Q water (resistivity 18.2 mΩ·cm at 25 °C).

### 3.2. Protein Solubility

The pea protein solubility was characterized at pH 3, 5 and 7 according to Bradford [43] with slight modifications. Protein dispersion (1.0% *w*/*w*) was kept under agitation for 1 h at ambient temperature, and then the samples were centrifugated at 10,000× *g* for 20 min at ambient temperature. In an assay tube, 100 µL of supernatant were mixed using a vortex with 2.5 mL of Bradford reagent. After 5 min, solubility was measured at 595 nm in a spectrophotometer UV/VIS model SP-220 (Biospectro/Merse, São Paulo, Brazil). A calibration curve was made with bovine serum albumin (R^2^ = 0.98).

### 3.3. Zeta Potential of Polymers

A Zetasizer Nano ZS (Malvern Instruments Ltd., Worcestershire, UK) was used to determine the zeta potential of the polymeric solutions (pH 2–7), and their mixtures at different PP:XG ratios (4:1, 8:1 and 12:1) and pHs below the isoelectric point of the protein, to promote electrostatic interaction. Measurements were made in triplicate at 25 °C in a DTS1070 capillary cell.

### 3.4. Emulsion Preparation

The emulsion and High Internal Phase Emulsion were prepared as previously described [26] with some modifications. Dispersions of PP and XG were prepared at 2.0% (*w*/*w*) in Milli-Q water containing 0.01% sodium azide to prevent microorganism growth. For complete hydration of the biopolymers, samples were allowed at room temperature and under mechanical stirring overnight. To promote the electrostatic deposition of XG in the PP layer at low pHs (layer-by-layer technique), the dispersions’ pH was adjusted at 3.0, 5.0 and 7.0 using phosphoric acid 0.66 M or NaOH 1.2 M. The 60/40 oil-in-water emulsions were obtained using an Ultraturrax^®^ (IKA^®^-Werke GmbH and Co. KG, Staufen im Breisgau, Germany). A primary emulsion was obtained, homogenizing SO with PP solution at 15,500 rpm by 6 minutes. Subsequently, the XG solution was added in a 4:1, 8:1 or 12:1 PP:XG ratios and the final emulsion was homogenized at 11,000 rpm for 3 min.

### 3.5. High Internal Phase Emulsion (HIPE) Preparation

All the emulsions were dried at 65 °C for 48 h using an ORION 515 oven (FANEM, São Paulo, Brazil). To obtain the HIPEs, the dried emulsion was manually homogenized, and the water loss was determined gravimetrically.

### 3.6. Emulsions and HIPEs Characterization

#### 3.6.1. Microstructure

A Carl Zeiss optical microscope (Axio Scope A1, Lower Saxony, Germany) was used to observe the microstructure of the emulsions and HIPEs at 10× amplitude. Optical and fluorescence microscopies were made, and the images were evaluated in the software AxioVision Rel. 4.8 (Carl Zeiss, Germany). To dye the sunflower oil (red) and the biopolymers (green), a drop of Nile red (0.1 g L^−1^ in polyethylene glycol) and a drop of FITC (0.2 g mL^−1^ in ethanol) (Sigma Aldrich, Wicklow, Ireland) were added to the emulsion or HIPE sample.

#### 3.6.2. Droplet Size

To determine the emulsions’ droplet mean diameter (D_[4,3]_ (Equation (1))), size distribution and span (Equation (2)), laser diffraction equipment (Mastersizer 2000, Malvern Instrument Ltd., Malvern, UK) was used. The larger peak’s Mode was determined based on the size distribution.

(1) $$D_{\left[4,3\right]}=\left(\sum n_{i}d_{i}^{4}\right)/\left(\sum n_{i}d_{i}^{3}\right)$$

(2) $$Span=\left(d_{\left(90\right)}-d_{\left(10\right)}\right)/d_{\left(50\right)}$$

where *d_i_* is the droplet diameter, *n* the number of drops and *d*_(10)_, *d*_(50)_, *d*_(90)_ are the diameters at 10%, 50% and 90% of cumulative volume, respectively.

To determine the droplet mean diameter of the HIPEs, a micrograph analysis was made using the Software ImageJ. The area (S) of 400 droplets was measured and the theoretical mean diameter (D_[Th]_) (Equation (3)) was calculated [44].

(3) $$D_{\left[\mathrm{Th}\right]}=\sqrt{4S/\pi }$$

#### 3.6.3. Rheological Assays

An AR1500ex rheometer (TA Instruments, Elstree, UK) was used to make the rheological tests for emulsions and HIPEs. A 40 mm flat plate geometry was used with a gap of 600 µm. Steady-shear flow curves were obtained in three cycles (up, down, up) in a range of 1–300 s^−1^, and the data were adjusted to the Herschel–Bulkley (HB) equation (Equation (4)). The linear viscoelastic region was determined in a range of 0.01–1000 Pa at 1 Hz of oscillatory stress. Frequency sweep (0.1–100 Hz) was made at 0.1 Pa of oscillatory stress to determine viscoelastic parameters. All measurements were made at 25 °C. Finally, a temperature sweep (25–80 °C) was made to determine structural changes due to thermal effects.

(4) $$\sigma =\sigma_{0}+k\cdot \gamma^{n}$$

where *σ* is the shear stress (Pa); *σ*_0_ is the initial shear stress (Pa); *k* is the consistency index (Pa·s), *γ* is the shear rate (1/s), and n is the flow behavior index (dimensionless).

#### 3.6.4. Oil Loss Determination

Approximately 1 g of emulsions or HIPE was disposed on Eppendorf tubes, and the oil loss was determined weekly, along a nine-week storage period at 5 and 25 °C. The samples were centrifuged at 10,000 rpm for 30 min, and the free oil was removed. The mass without free oil was weighed, and the oil loss was determined (Equation (5)).

(5) $$Oil\;loss=\left(m_{i}-m_{f}\right)/\left(m_{i}-m\right)\times 100$$

where $m_{i}$ is the mass of the initial sample and the Eppendorf $m_{f}$ is the mass of Eppendorf with sample after centrifugation and *m* is the mass of the Eppendorf.

### 3.7. Statistical Analysis

All experiments were performed in triplicate and the results were statistically analyzed using Microsoft Excel software (2010). The difference between the means was evaluated using the Tukey test (*p* ≤ 0.05).

## 4. Conclusions

This study presents the sunflower oil structuring using emulsification as an indirect method to obtain stable systems for 9 weeks. Pea protein (PP) and xanthan gum (XG) were used as structurants of emulsions (60% of oil) and High Internal Phase Emulsions (HIPEs, Oil concentration > 73%) varying the pH (3, 5 and 7) and the PP:XG ratio (4:1, 8:1 and 12:1). Each system has specific macroscopic and rheological properties that can be exploited depending on the matrix where it will be applied (food or cosmetic approach), especially the HIPEs systems, presenting a creaming structure and a typical week gel-like behavior with different viscoelastic values. The higher the pea protein: xanthan gum ratio and pH, the higher the viscoelasticity of the system. Based on the results of storage stability at 5 and 25 °C, emulsions obtained at pHs 5 and 7 in the ratios PP:XG 4:1, 8:1 and 12:1, as well as that obtained at pH 3 in the ratio 4:1, were stable to the oil release during the studied storage time. For HIPEs, those obtained at pH 7 in the ratios PP:XG 4:1, 8:1 and 12:1, and at pH 5 at PP:XG ratio 4:1 showed the lowest oil release after 9 weeks of storage. The information generated in this work allows the development of a product that can be used to produce a wide range of food formulations with lower fat content in their composition or as a vehicle of lipophilic bioactive compounds.

## Figures and Tables

**Table 1:** Mass balance of HIPEs after emulsion drying (100 g of initial emulsion).

Ratio	pH	Mass Loss (g)	Total Mass (g)	Water		Oil		PP + XG	
				g	%	g	%	g	%
4:1	3	33.33 ± 0.59 ^Ab^	66.67	5.87	8.80	60.00	90.00	0.80	1.20
	5	17.38 ± 0.80 ^Cc^	82.62	21.82	26.41	60.00	72.62	0.80	0.97
	7	22.38 ± 0.36 ^Bc^	77.62	16.82	21.67	60.00	77.30	0.80	1.03
8:1	3	37.28 ± 0.40 ^Aa^	62.72	1.92	3.06	60.00	95.66	0.80	1.28
	5	23.17 ± 1.76 ^Cb^	76.83	16.03	20.86	60.00	78.09	0.80	1.04
	7	29.83 ± 1.05 ^Bb^	70.17	9.37	13.35	60.00	85.51	0.80	1.14
12:1	3	37.96 ± 1.87 ^Aa^	62.04	1.24	2.00	60.00	96.71	0.80	1.29
	5	26.38 ± 1.03 ^Ca^	73.62	12.82	17.41	60.00	81.50	0.80	1.09
	7	31.26 ± 0.90 ^Ba^	68.74	7.94	11.55	60.00	87.29	0.80	1.16

PP: pea protein, XG: xanthan gum. Different letters indicate significant difference (*p* < 0.05) for mass loss. Capital letters: difference in the same ratio. Lowercase letters: difference in the same pH.

**Table 2:** Droplet mean diameter (D_[4,3]_, D_[Th]_), mode (Mo) and Span of the emulsions and HIPEs.

		Emulsions			HIPEs			
Ratio	pH	D_[4,3]_ (µm)	Mo (µm)	Span	D_[4,3]_ (µm) *	D_[Th]_ (µm)	Mo (µm) *	Span *
4:1	3.0	34.28 ± 0.89 ^Bc^	36.29 ± 1.77 ^Bb^	1.42 ± 0.04	--	--	--	--
	5.0	51.79 ± 7.20 ^Aa^	43.96 ± 1.99 ^Aab^	1.51 ± 0.23	27.16	20.38 ± 6.22	22.44	1.05
	7.0	19.17 ± 0.65 ^Cb^	16.21 ± 0.79 ^Cb^	1.57 ± 0.11	16.33	11.46 ± 2.83	10.02	0.80
8:1	3.0	51.95 ± 2.16 ^Ab^	55.35 ± 2.50 ^Aa^	1.24 ± 0.05	--	--	--	--
	5.0	43.61 ± 1.9 ^Ab^	45.68 ± 2.23 ^Ba^	1.21 ± 0.08	25.38	18.99 ± 5.39	15.88	0.91
	7.0	18.75 ± 0.52 ^Bb^	15.60 ± 0.71 ^Cb^	1.71 ± 0.04	10.86	8.74 ± 2.30	7.96	0.89
12:1	3.0	59.56 ± 1.42 ^Ca^	57.51 ± 2.81 ^Aa^	1.17 ± 0.08	--	--	--	--
	5.0	40.60 ± 1.19 ^Bb^	41.53 ± 2.51 ^Bb^	1.28 ± 0.06	31.25	16.60 ± 6.81	12.62	1.48
	7.0	23.16 ± 1.04 ^Aa^	18.55 ± 1.12 ^Ca^	1.61 ± 0.13	10.71	8.00 ± 2.42	7.86	1.01

Different letters indicate significant difference (*p* < 0.05) in the same column. * Parameters calculated from the Dth values of the droplet size distribution.

**Table 3:** Storage (G′) and loss modulus (G″), limiting value of oscillatory stress (OS_L_), loss-tangent (tan δ), flow-point stress (FP_S_) and flow-point G (FP_G_) for emulsions and High Internal Phase Emulsions (HIPEs) produced at different pea protein: xanthan gum ratios (PP:XG) and pHs.

	PP:XG	pH	G′_LVR_ (Pa)	G″_LVR_ (Pa)	OS_L_ (Pa)	Tan δ_LVR_	FPs (Pa)	FP_G_ (Pa)
Emulsions	4:1	3	236 ± 4 ^Ab^	43 ± 1 ^Aa^	0.71 ± 0.08	0.19 ± 0.01	22.52 ± 2.58	22 ± 1
		5	185 ± 6 ^Ba^	41 ± 2 ^Aa^	3.57 ± 0.41	0.22 ± 0.01	17.90 ± 2.05	38 ± 4
		7	144 ± 2 ^Ca^	32 ± 1 ^Ba^	1.79 ± 0.20	0.22 ± 0.01	17.90 ± 2.05	21 ± 4
	8:1	3	253 ± 2 ^Aa^	40 ± 2 ^Aa^	2.17 ± 0.23	0.16 ± 0.01	62.99 ± 0.03	24 ± 1
		5	140 ± 1 ^Bb^	31 ± 2 ^Bb^	1.86 ± 0.18	0.23 ± 0.01	14.22 ± 1.63	23 ± 6
		7	98 ± 2 ^Cc^	31 ± 2 ^Ba^	0.71 ± 0.08	0.32 ± 0.02	7.94 ± 0.00	21 ± 1
	12:1	3	253 ± 2 ^Aa^	38 ± 1 ^Aa^	2.72 ± 0.28	0.15 ± 0.01	50.10 ± 0.01	26 ± 3
		5	128 ± 1 ^Bc^	26 ± 1 ^Bc^	1.42 ± 0.16	0.20 ± 0.01	17.89 ± 2.05	33 ± 9
		7	113 ± 2 ^Cb^	22 ± 1 ^Cb^	0.90 ± 0.20	0.19 ± 0.01	9.99 ± 0.01	20 ± 1
HIPEs	4:1	5	311 ± 2 ^Bc^	48 ± 2	5.66 ± 0.65	0.15 ± 0.01	50.11 ± 0.01	108 ± 6
		7	726 ± 4 ^Ac^	79 ± 4	2.25 ± 0.26	0.11 ± 0.01	60.65 ± 5.15	107 ± 8
	8:1	5	323 ± 1 ^Ba^	54 ± 3	3.57 ± 0.41	0.17 ± 0.01	56.60 ± 6.49	78 ± 6
		7	1183 ± 3 ^Aa^	105 ± 6	11.29 ± 1.29	0.09 ± 0.01	--	--
	12:1	5	319 ± 1 ^Bb^	43 ± 1	4.50 ± 0.51	0.13 ± 0.01	86.28 ± 9.14	76 ± 3
		7	1035 ± 3 ^Ab^	90 ± 6	14.22 ± 1.63	0.08 ± 0.01	--	--

FP: flow-point (G′ = G″), linear viscoelastic region (LVR).

## Data Availability

Not applicable.

## Sample Availability

Samples of the compounds are not available from the authors.
